# Supplementary material for: Consistent Biomarkers and Related Pathogenesis Underlying Asthma Revealed by Systems Biology Approach
Source: Int J Mol Sci. 2019 Aug 19;20(16):4037. doi: 10.3390/ijms20164037 (PMC6720652; doi:10.3390/ijms20164037)
Supplement: Supplementary file 1 [file ijms-20-04037-s001.zip › ijms-566768-supplementary-XML/Supplementary Material S2-Reports of Quality control for ten datasets.zip!/Supplementary Material S2-Reports of Quality control for ten datasets/QC_report_for_GSE63142/ma.pdf]

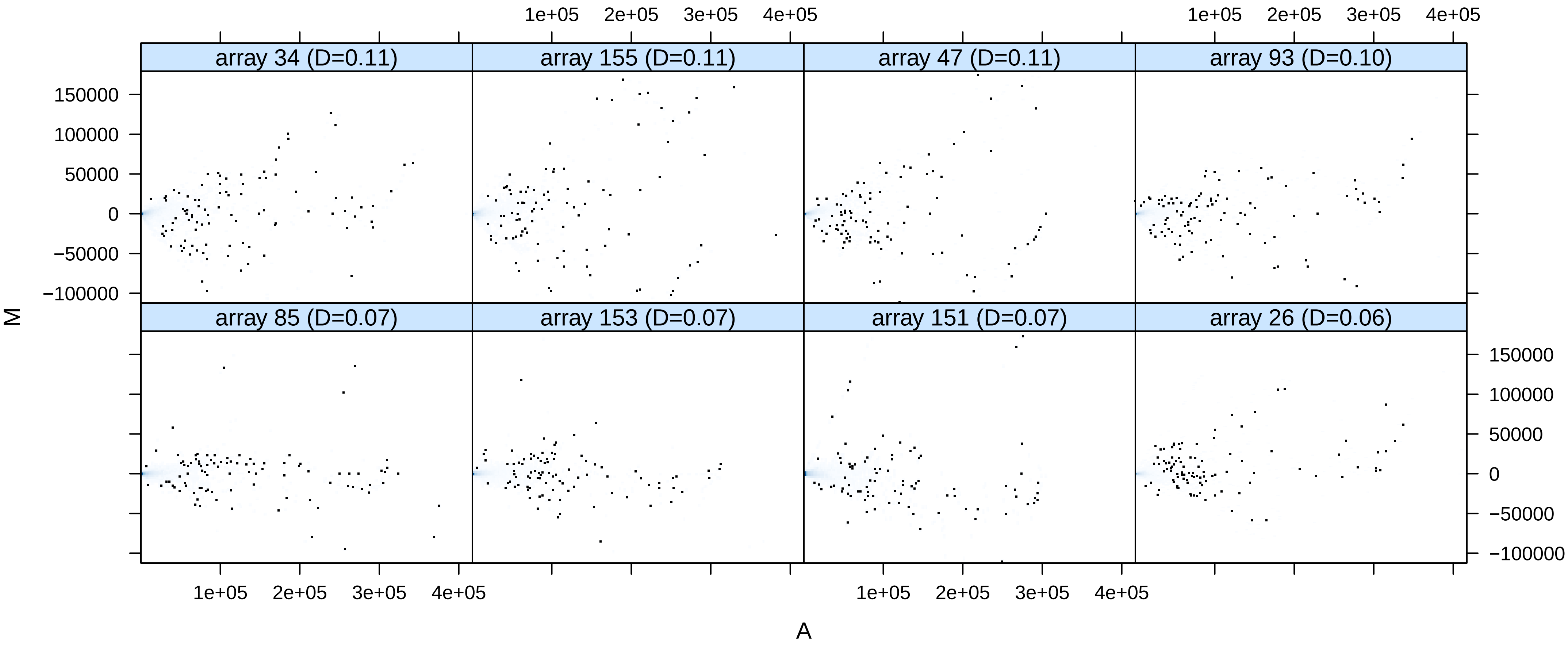
array 34 (D=0.11)
array 155 (D=0.11)
array 47 (D=0.11)
array 93 (D=0.10)
array 85 (D=0.07)
array 153 (D=0.07)
array 151 (D=0.07)
array 26 (D=0.06)
M
A
1e+05
2e+05
3e+05
4e+05
150000
100000
50000
0
−50000
−100000
